# MUC1 and E-cadherin immunohistochemistry of endometrium cannot predict the outcome of *in vitro* fertilization: A case-control study

**DOI:** 10.12688/f1000research.17929.1

**Published:** 2019-02-06

**Authors:** Saumitra Chakravarty, Mohammed Kamal

**Affiliations:** 1 Department of Pathology, Bangabandhu Sheikh Mujib Medical University, Shahbag, Dhaka, 1000, Bangladesh

**Keywords:** MUC1, E-cadherin, IVF, IHC

## Abstract

**Background:** Although *in vitro* fertilization (IVF) has played a major role in the management of infertility, its failure rate is still 60-80% and most of the causes failure are unknown. Therefore, a histomorphology-based predictive tool to forecast IVF outcome that utilizes expression data of certain cellular adhesion molecules in endometrium pertaining to successful implantation might provide the theoretical basis to develop a low-cost laboratory investigation suited for low to middle income countries as opposed to the expensive gene expression based tools like endometrial receptivity array. In this study, mucin 1 (MUC1) and E-cadherin immunohistochemistry of endometrium from aspiring IVF women were analyzed to see if there is any correlation between signal intensities and endometrial receptivity in terms of IVF outcome.

**Methods:** This was a case-control study conducted among women of reproductive age with infertility who underwent IVF at the Centre for Assisted Reproduction (CARe), Dhaka between March and December 2017. Endometrial biopsy samples were collected and routine histological as well as immunohistochemical analysis was performed on those samples at the Department of Pathology, Bangabandhu Sheikh Mujib Medical University (BSMMU), Dhaka. A total of 21 patients, 17 cases (IVF failure) and four controls (IVF success), were included in the study by consecutive convenient sampling. Relevant history and medical records of each of the patients were also obtained accordingly.

**Results:** No statistically significant correlation was found between IVF outcomes and the signal intensities in endometrium produced by MUC1 and E-cadherin immunohistochemistry.

**Conclusions:** Despite the fact that this study did not find any statistically significant correlation between endometrial immunohistochemistry of MUC1 and E-cadherin and IVF outcome, further studies may incorporate gene expression arrays to supplement or revise those findings.

## Introduction

Although birth control measures are highlighted as one of the major reproductive health concerns in third world countries, the population suffering from infertility often goes unnoticed. Globally, 12.4% of women who are trying to conceive in the age range of 20–44 years are infertile, accounting for both primary and secondary infertility; south Asia is one of regions with the highest prevalence of infertility ^1^. Despite the fact that about 16,700 infertility cases are enrolled annually in the handful number of facilities available in Bangladesh and many more of the cases remain unreported, the medical, social, financial and psychological burden associated with the condition is almost always under-appreciated in health statistics ^2^. There is no account of how many infertile couples seek help from fraudulent and pseudoscientific agencies in the country, which may add significantly to the burden ^3^. The high cost of infertility treatment happens to be a major barrier for aspiring couples to seek medical help in Bangladesh ^2^. Failure rates as high as 60%, even in the most developed countries, make the application of *in vitro* fertilization (IVF) and other assisted reproductive technologies an expensive gamble ^4^. Moreover, the cause of infertility remains unexplained in 10.95% of cases ^2^.

To find a solution to this problem, a research group in Spain analyzed mRNA from endometrial biopsy samples of expecting women and identified 238 genes that play a crucial role in implantation ^5^. Using the results, they developed a customized mRNA array and bioinformatic tool for testing expression of those 238 genes to predict not only the possibility of implantation failure but also the personalized window of implantation (WOI) during which the chance of a successful implantation is maximum. That is called endometrial receptivity array (ERA). As good as it may sound, ERA is not without its own shortcomings. Firstly, it is quite expensive ^6^. Secondly, availability is limited, only at a handful of institutes in Spain, Japan, the USA and a few other countries. Thirdly and most importantly, even if we can improve the odds for success by analyzing all 238 genes’ expression, the results are not translatable in terms of targeted therapy of infertility because there are simply too many targets. Despite these limitations, there has been no other tool as reliable and precise as ERA to date to predict and improve IVF outcome (i.e., success/failure) ^7^. Therefore, efforts are being made to discover clever work-arounds to cut both the cost and number of targets with little compromise in terms of predictive values. ERA-directed proteomic studies of uterine biopsies from both groups of women (with implantation success and failure) indicate that secretory endometrium expresses certain cellular adhesion molecules that firmly attach the blastocyst at the site of implantation ^8^. This cell-cell interaction is pivotal not only for successful implantation but also for maintenance of pregnancy. Proteomics provide a list of few pathways that have a handful of protein molecules ^9^. These are the key players of implantation. So far, five to ten proteins, including mucin 1 (MUC1) and E-cadherin, have shown potential to constitute a panel for predictive screening of implantation failure ^10^.

The peculiarity of MUC1 is that it acts as a ‘guiding molecule’ for the blastocyst during implantation in order to direct it to the implantation site of the decidua ^8^. In receptive endometrium, MUC1 expression is generally high, especially in the endometrium during secretory phase, compared to non-receptive endometrium ^11, 12^. Interestingly, there is always a small patch of decidua where the receptive endometrium almost completely lacks MUC1 expression during the WOI and that marks the site of implantation. Hence the connotation of a ‘guiding molecule’, as if the blastocyst wanders about the decidua until it finds the designated spot to implant marked by the absence of MUC1 ^8, 13^. In case of non-receptive endometrium as in IVF failures or recurrent spontaneous abortions, global down-regulation of MUC1 throughout the whole of the endometrium fails to elicit the appropriate signal for implantation. However, it should be noted that if implantation is successful then MUC1 expression is quickly depleted and therefore it is one of the negative stains in normal placenta ^14^.

Cadherins constitute a group of glycoproteins responsible for the calcium-dependent cell-cell adhesion mechanism. It has been postulated that E-cadherin exhibits dual attributes consisting of an increased expression during secretory phase and early implantation: epithelial cell adhesiveness by E-cadherin is controlled by intracellular calcium and rising progesterone levels induce calcitonin expression and thus increase the concentration of intracellular calcium, which then suppresses E-cadherin expression at cellular contact sites. It corresponds to the adhesiveness of decidua-blastocyst interface while a subsequent spell of its down-regulation corresponds to epithelial dissociation and trophoblastic invasion ^8^. Although cyclical variation in mRNA levels of E-cadherin is not reflected at protein level as detected by IHC, there are indeed immunohistochemically detectable differences of E-cadherin expression between receptive and non-receptive endometrium ^15^. Interestingly, progesterone level has somewhat inverse relationship with E-cadherin expression ^16, 17^. Moreover, it has been demonstrated that targeted mutations in E-cadherin gene are associated with implantation failure ^18^.

We sought to evaluate in this study if there is any correlation between IVF outcome and semi-quantitative immunohistochemistry scores (H-score) of MUC1 and E-cadherin in endometrial epithelium and stroma.

## Methods

### Study design

The present study was a case-control study. The study subjects, i.e., all female patients attending Centre for Assisted Reproduction (CARe), Dhaka, Bangladesh who fulfilled the selection criteria, including giving informed written consent to participate in the study, between 1 ^st^ March and 31 ^st^ December 2017 were sorted into either case or control groups according to the selection criteria. The key outcome variable that distinguished between the two groups was post-IVF pregnancy status. If the status was non-pregnant then the subject was included in the case group (n=17) and if the participant was pregnant then she was included in the control group (n=4). The sample size was estimated using Kesley’s formula for case-control study ^19^. All other criteria being adequately fulfilled as well as normalizing for possible confounders, e.g. age, body mass index (BMI), obstetric history, menstrual history, contraceptive history, previous IVF outcome, follicular stimulating hormone (FSH) level and luteinizing hormone (LH) level.

Endometrial biopsy samples collected from both groups prior to IVF procedure, preserved as formalin-fixed paraffin embedded (FFPE) blocks in the archive, were assessed both by routine histology and immunohistochemically to determine the values of key exposure variables, i.e., endometrial dating and immunohistochemical H-scores for MUC1 and E-cadherin stains. Those values were used to determine if the exposure variables correlated to the outcome variable, which can predict the outcome of IVF within statistical limits.

### Selection criteria

**Inclusion criteria for cases**

1. Selected as a candidate for IVF by competent physician.
2. Provided informed written consent to participate in the study.
3. Underwent endometrial biopsy according to the protocol mentioned in data collection procedure.
4. Post-IVF pregnancy status indicates IVF failure.

**Inclusion criteria for controls**

1. Selected as a candidate for IVF by competent physician.
2. Provided informed written consent to participate in the study.
3. Underwent endometrial biopsy according to the protocol mentioned in data collection procedure.
4. Post-IVF pregnancy status indicates IVF success.

**Exclusion criteria for both cases and controls**

1. Refusal to participate in the study or withdrawal from the study at any point.
2. If it is not possible or contra-indicated to obtain endometrial biopsy at all or at the designated time as mentioned in data collection procedure.
3. If the performance of IVF is cancelled or postponed beyond the specified duration of study.
4. If post-IVF pregnancy status could not be ascertained within the specified duration of study or the patient is dropped out from follow-up.
5. Co-morbid conditions like tuberculosis, endometrial hyperplasia, HIV/AIDS, thyroid diseases, diabetes mellitus, hypertension, immunological diseases etc.
6. Incomplete medical record or inadequate sample.

### Data collection procedure

***Procedures performed at CARe*.** Endometrial scratching was the method used to collect endometrial biopsy sample. The procedure was performed by a competent physician on the 21 ^st^ day of the natural cycle or on a day accordingly adjusted for irregular, longer or hormone-assisted cycle immediately before the commencement of IVF cycle. The samples were transferred immediately to 10% buffered formalin in a properly labeled container. The container was then kept at room temperature for 24 hours before it was transported to Bangabandhu Sheikh Mujib Medical University (BSMMU) where the laboratory procedures commenced. It was imperative not to freeze the sample because that would cause freezing artifacts rendering it unsuitable for subsequent histological procedures as well as microscopy.

Informed written consent was taken prior to the biopsy procedure. Serum levels of FSH and LH were measured on day 3 of the cycle immediately prior to the IVF cycle. Relevant history, clinical information and most of the investigation findings featuring the key variables were collected by the attending physician or the primary investigator via a pre-formed questionnaire at the time of taking the biopsy. Embryo was transferred on the next suitable WOI as determined by the conventional IVF protocol, usually after 72 hours following the oocyte retrieval during which the fertilization took place *in vitro*.

Post-IVF pregnancy check was performed by measuring serum human chorionic gonadotropin (hCG) level after two weeks of embryo transfer, taking into account the iatrogenic effect of hCG which was being administered during that period of time. The pregnancy status was recorded accordingly. The rest of the relevant investigation findings unavailable during collection of endometrial biopsy were then recorded. In the case of irregular menstrual cycle, the consultant physician determined the adjusted dates of IVF protocol according to the patient’s previous cycles over one year along with the findings from transvaginal sonography. Table 1 summarizes the IVF protocol that was followed along with the various points in data or sample collection for this study.

**Table 1.** Summary of the *in vitro* fertilization protocol.

Cycle	Day	Procedure/ investigation	Data/ sample collected
Non-transfer	3	• Serum FSH • Serum LH	• FSH level • LH level • FSH:LH ratio
	12	• Transvaginal sonography • Endometrial scratch (of four patients)	• Endometrial biopsy • History taking and anthropometry
	20–23	• Endometrial scratch (of 17 patients) • GnRH administration	• Endometrial biopsy • History taking and anthropometry
Transfer	2	• GnRH administration • Combined preparation of FSH and LH (1:1) administration	–––
	14–15	• Oocyte retrieval • hCG administration • Semen collection (from husband)	–––
	14–17	Fertilization of oocyte with spermatozoa *in vitro*	–––
	17–18	Embryo transfer	–––
Post-transfer	ET+14	Serum β-hCG	Pregnancy status

ET, embryo transfer; FSH, follicle stimulating hormone; GnRH, gonadotropin releasing hormone; hCG, human chorionic gonadotropin; IVF, *in vitro* fertilization; LH, luteinizing hormone.

***Procedures performed at BSMMU*.** Routine tissue processing was done followed by preparation of FFPE blocks for every sample, one for each patient. Hematoxylin-Eosin (HE) stained permanent slides were prepared from those FFPE blocks. Routine microscopy of the HE slides was performed to look for the possibility of tuberculosis, hyperplasia and malignancy as well as for the estimation of endometrial date. After receiving information regarding post-IVF pregnancy status from CARe Hospital, and also combining the information gathered from routine HE histology as well as the data collection sheet, each sample was either included in the study as a case or a control according to inclusion criteria, or excluded from the study according to exclusion criteria. After the sorting of the patients into case and control groups according to the selection criteria, a computer-aided randomized patient coding technique was applied to ensure double blind design so that neither the investigator, nor the supervisor or the personnel directly involved in the study can know which patient or slide belonged to which group until the master datasheet is prepared.

FFPE blocks of cases and controls both were then used for preparation of immunohistochemistry slides, by staining each section with antibodies ( Table 2). Tissue microarray technique was used for the purpose to reduce the expenditure.

**Table 2.** Summary of immunohistochemistry staining of the slides.

Antibody	Clone/Catalogue number and manufacturer	Antigen retrieval		Dilution	Positive control
		Buffer pH	Heating method		
MUC1/EMA	MO613, Dako	9	Automated (65°C-97°C -65°C cycle; 20 minute)	RTU	Skin (epidermis)
E-cadherin	IS059, Dako	9	Automated (65°C-97°C -65°C cycle; 20 minute)	RTU	Normal breast tissue

RTU, ready to use.

Antigen retrieval was performed according to the manufacture’s manual that comes with the designated IHC markers. Endogenous peroxidase activity was blocked by 3% hydrogen peroxide for 20 minutes. Slides were then incubated with appropriate clone of designated antibodies at suitable dilutions. Then all the slides underwent the same protocol: slide reagents A (Polymer Helper) and B (polyperoxidase anti-mouse IgG) were used for the reaction and the slides were counterstained with Mayer’s hematoxylin followed by staining with 3,3’-diaminobenzidine.

**Figure 1.**
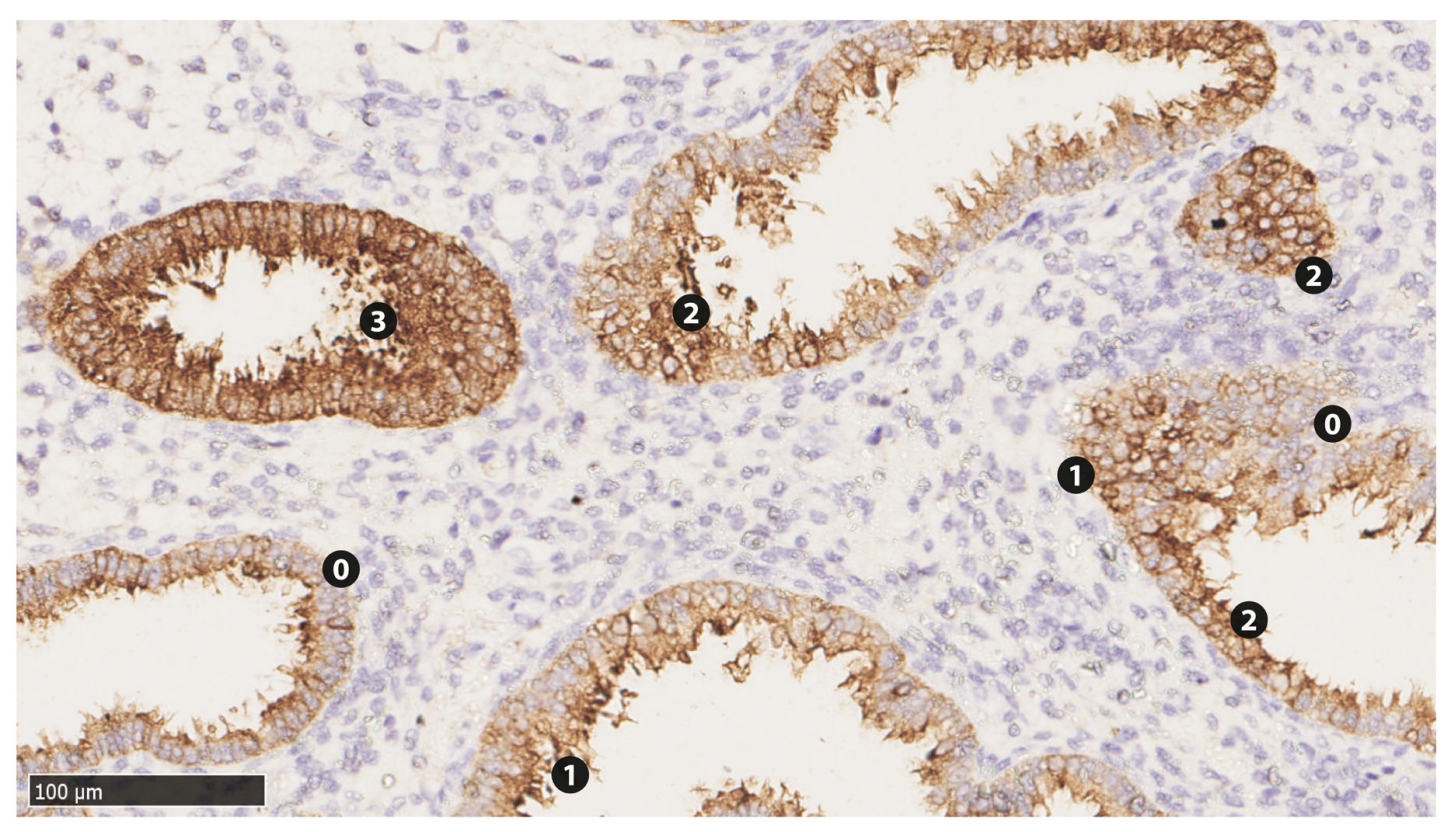
Immunohistochemical staining of MUC1 in endometrial tissue collected from patient C3 at her 21 ^st^ day of non-transfer cycle (x200). Numbered labels are placed adjacent to the epithelia corresponding to their respective relative intensities ( Table 3). Stromal cells show lack of staining.

The slides were then evaluated in terms of H-score by two Pathology consultants independently to rule out inter-observer variation. H-score takes into account two aspects of cellular staining by immunohistochemical markers: relative intensity (RI) and percentage of cells (%C) stained. RI is ranked 0–3 as follows: 0, no/ negative/ background staining; 1, weak positive; 2, strong positive; 3, very strong positive. In a given section, the percentage of cell (%C) that fits each of the four RI ranks are also estimated. Then the RI is multiplied by the corresponding %C value. Lastly, four of such products, one for each RI, are added up to get the H-score of that sample for a given marker ^9, 20^. Figure 1 and Table 3 show an example of the scoring system.

**Table 3.** Calculation of the H-score for MUC1 in endometrial tissue collected from patient C3 at her 21 ^st^ day of non-transfer cycle.

Patient code	Relative intensity (RI)	Percentage of cells (%C)	RI × %C	H-score = Σ(RI × %C)
C3	No/ negative/ background staining (0)	9	0	174
	Weak positive (1)	22	22	
	Strong positive (2)	55	110	
	Very strong positive (3)	14	42	

All of the slides were scanned by Hamamatsu nanoZoomer® slide scanner to create digitally accessible virtual slides for archival purpose. Microsoft Office Excel 2010 was used to perform statistical analysis ^19– 22^. For all statistical tests, p-value <0.05 was considered as statistically significant.

## Results and discussion

Results are prepared in four steps. Firstly, sorting and matching of case and control groups are done for probable confounding variables. Secondly, routine histomorphological evaluation of endometrial biopsy samples was done which includes endometrial dating. Thirdly, immunohistochemical markers in endometrial biopsy samples were evaluated with H-scores and optimum cutoffs were estimated. Lastly, correlations of immunohistochemical markers with IVF outcomes were calculated and statistical inferences were made. In summary, correlations between IVF outcome and H-scores of immunohistochemical markers MUC1 and E-cadherin are weakly positive and statistically insignificant.

Out of 21 patients, 17 patients (81%) had IVF failure (not pregnant) and four patients (19%) had IVF success (pregnant). After case-control categorization, the former group was considered case and the latter group was control. All the participants were from middle class to upper middle class according to socioeconomic strata. The women of the case group had a mean age of 34.00 ± 2.31 years while the mean age of the control group was 33.75 ± 4.79 years; the difference between the two groups were not statistically significant. There were no significant difference between them in terms of BMI, FSH, LH, FSH:LH ratio, menstrual and obstetric history which were considered to be the potential confounding factors. Their husbands were all within normal range of relevant reproductive parameters. None of the patients used any contraceptive method ever, except barrier method, and actively trying to conceive for at least two years. Degree of expression of MUC1 and E-cadherin as detected by immunohistochemistry was considered to be the exposure (H-scores ranging from 0-300) and post-IVF pregnancy status was considered as the outcome (pregnant or non-pregnant) in the present study.

**Figure 2.**
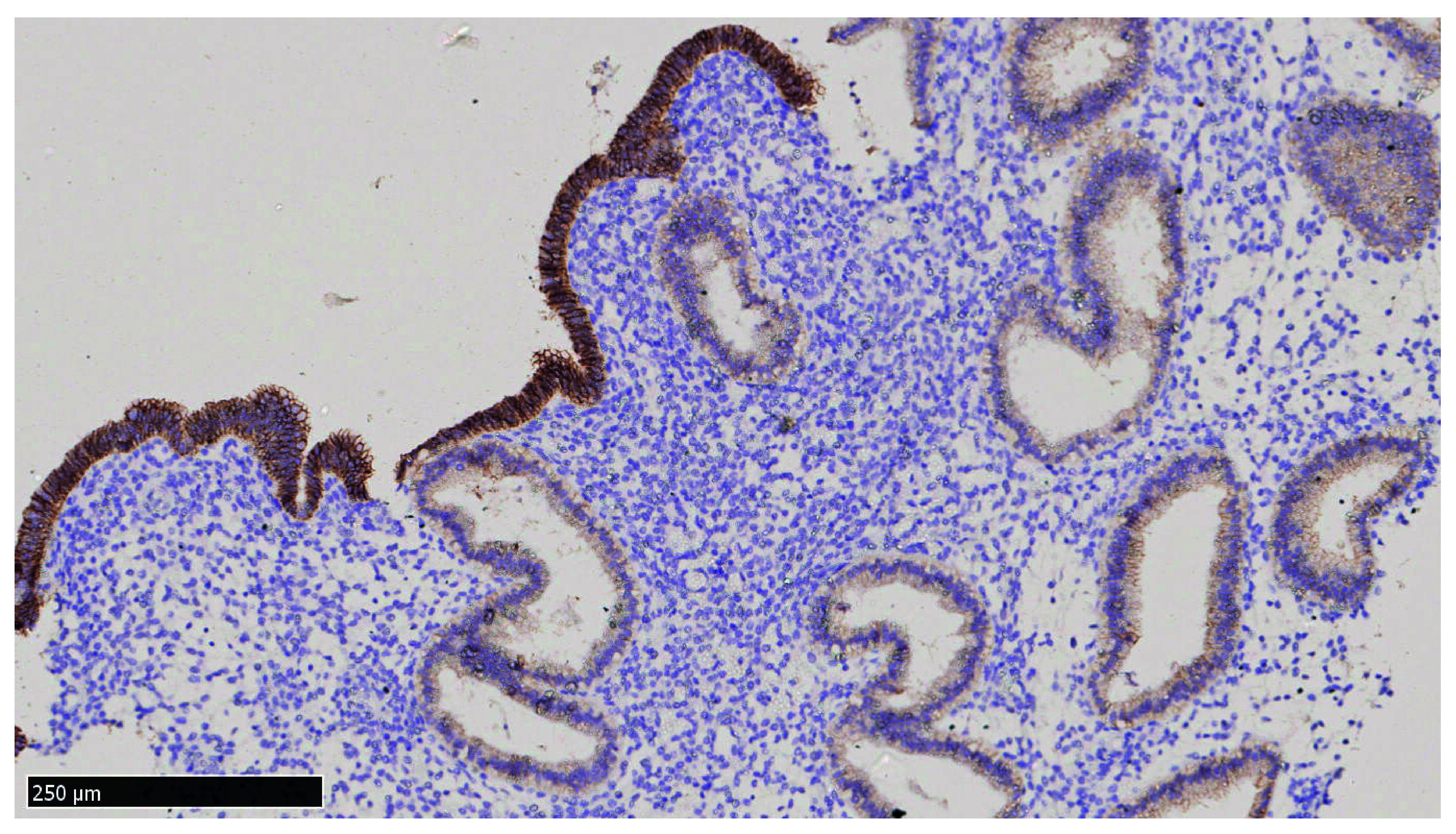
Immunohistochemical staining of E-cadherin in endometrial tissue collected from patient C6 at her 21 ^st^ day of non-transfer cycle where surface epithelia stained more intensely than the glandular epithelia beneath it (x100).

It is interesting to note that a peculiar staining pattern was observed with E-cadherin where the surface epithelia stained with markedly greater intensity than the glandular epithelia ( Figure 2). Most of the samples exhibit similar staining pattern with E-cadherin.

Comparison between case and control groups by unpaired t-test show statistically non-significant difference for mean H-scores of MUC1 as well as E-cadherin although MUC1 has a larger effect size (0.91) compared to that of E-cadherin (0.69). Calculation of inter-observer variation between two independent observers (Pathology Consultants) was measured on 10 randomly chosen tissue sections out of 21 samples by Chronbach’s alpha correlation technique ^21^. Inter-observer variation was found to be within acceptable limits for each of the stains ^22^.

Due to the variability of data obtained from two subsets of samples, namely, collected before 20 ^th^ day and collected on or after 20 ^th^ day, the statistical analysis for derivation of the cutoff scores were calculated separately for the two subsets on each of the markers. At every attempt to find the optimum cutoff, a receiver-operator characteristics (ROC) curve was drawn first and then its corresponding Youden’s indices (specificity + sensitivity – 1) were plotted if the ROC curve had an area under curve (AUC) greater than zero. The point with the maximum value of the Youden’s index was chosen as the optimum cutoff because it maximized the specificity and sensitivity of the marker in question ^23^. Instances where ROC curve had an AUC of zero unit, Youden’s index could not be meaningful, thus yielding the corresponding cutoff value undefined. AUCs of ROC curves were calculated using trapezoid rule and maximum Youden’s indices were obtained by direct measurement.

**Figure 3.**
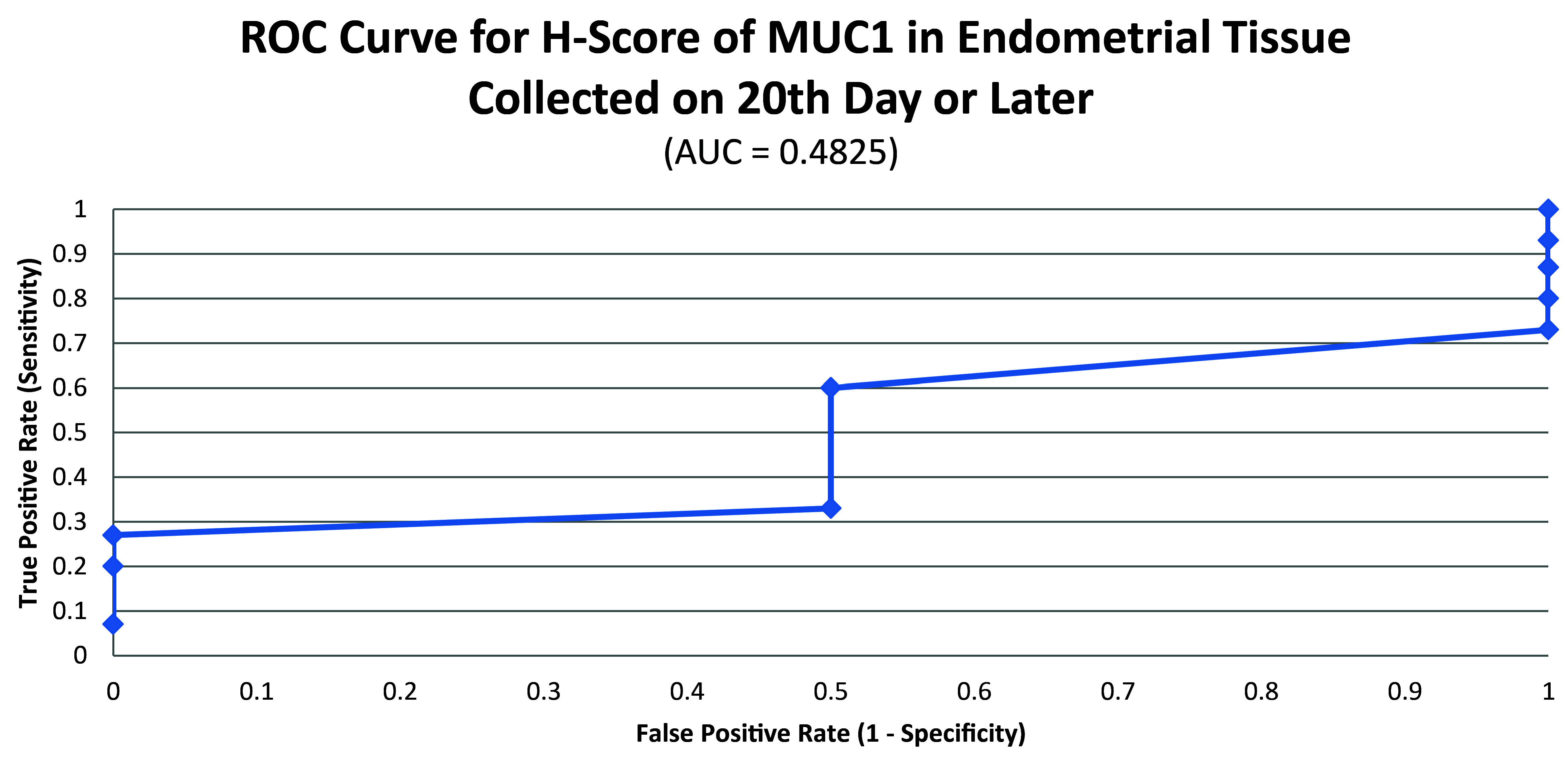
ROC curve for H-score of MUC1 in endometrial tissue collected on 20 ^th^ chronological day or later.

**Figure 4.**
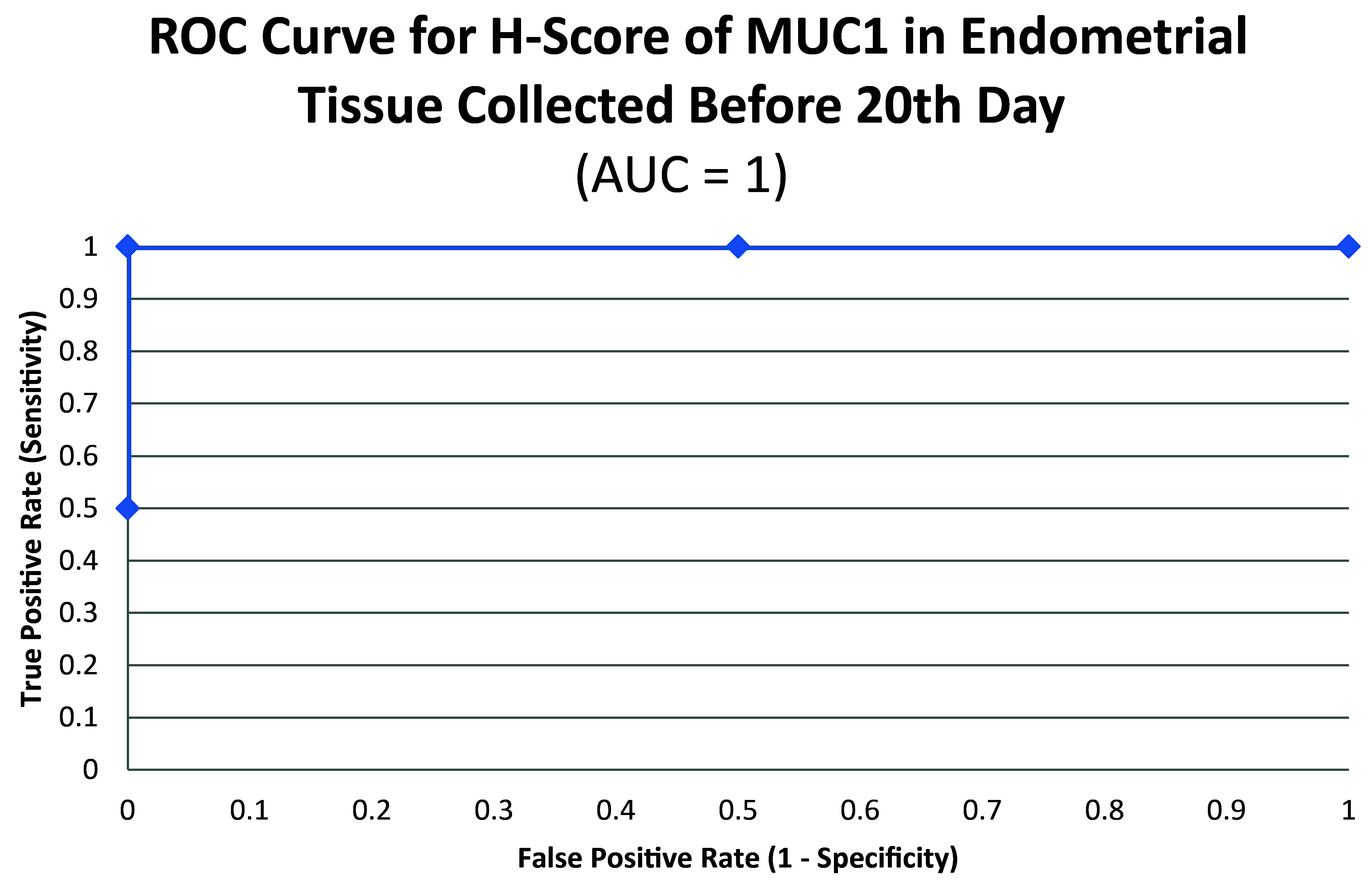
ROC curve for H-score of MUC1 in endometrial tissue collected before 20 ^th^ chronological day.

**Figure 5.**
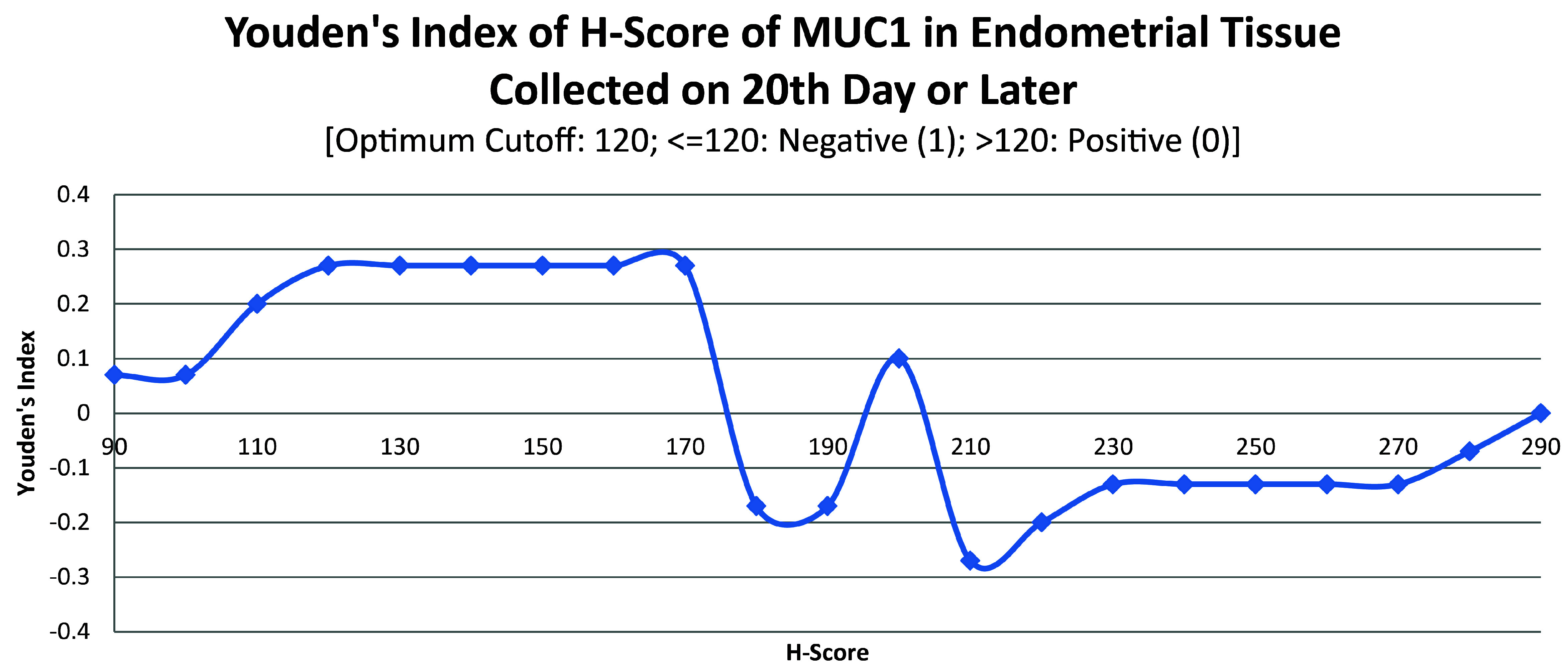
Youden’s index curve for H-score of MUC1 in endometrial tissue collected on 20 ^th^ chronological day or later.

**Figure 6.**
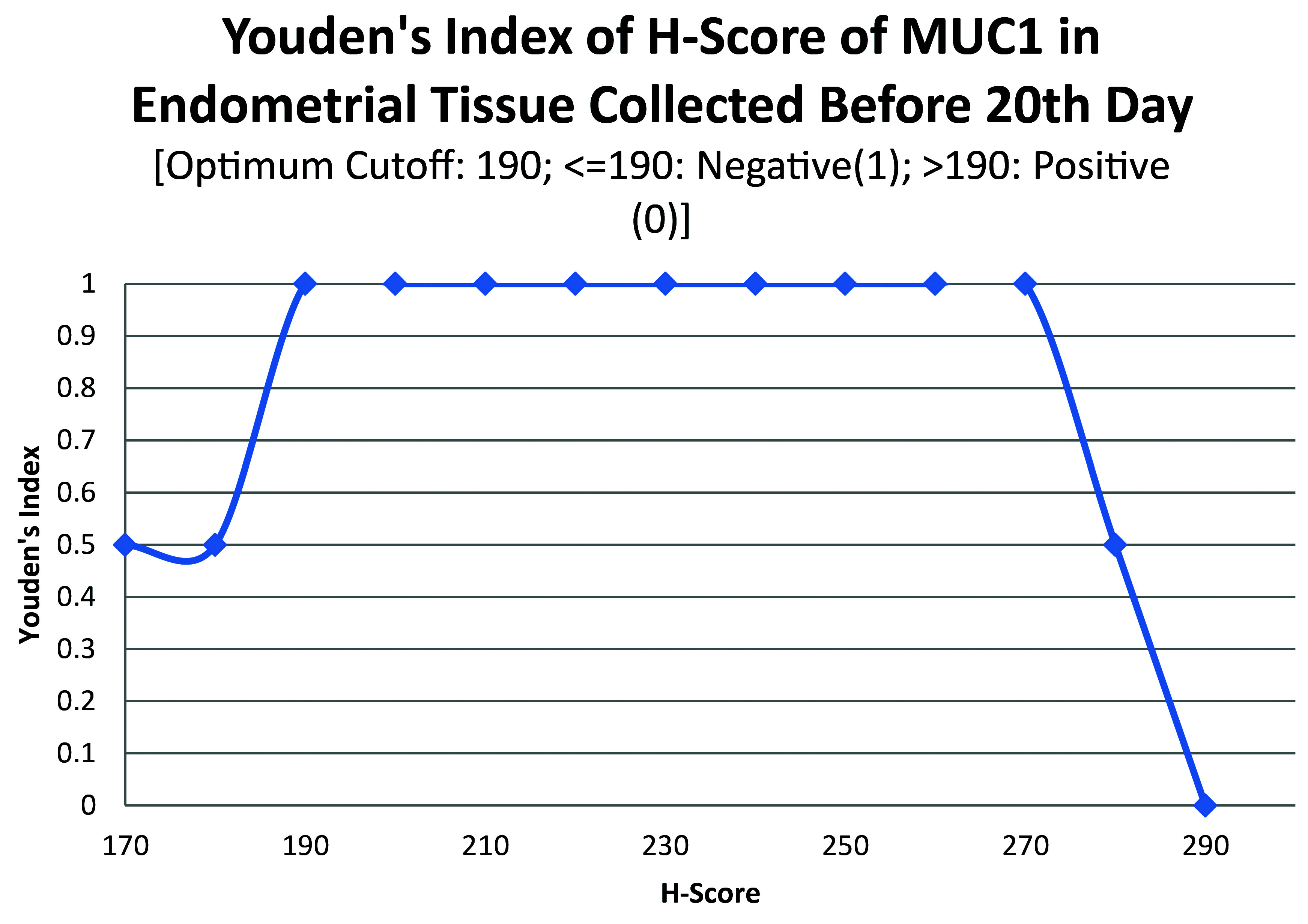
Youden’s index curve for H-score of MUC1 in endometrial tissue collected before 20 ^th^ chronological day.

ROC curve for MUC1 suggested the existence of optimum cutoff values for samples collected before, on or after the 20 ^th^ chronological day since AUC>0 held for all the instances ( Figure 3 and Figure 4). The cutoff for samples collected on or after 20 ^th^ day was 120 ( Figure 5) and the rest of the samples yielded a cutoff of 190 ( Figure 6). MUC1 status is to be considered negative if the H-score is less than or equal to the cutoff; if it is above the cutoff then the status is positive.

**Figure 7.**
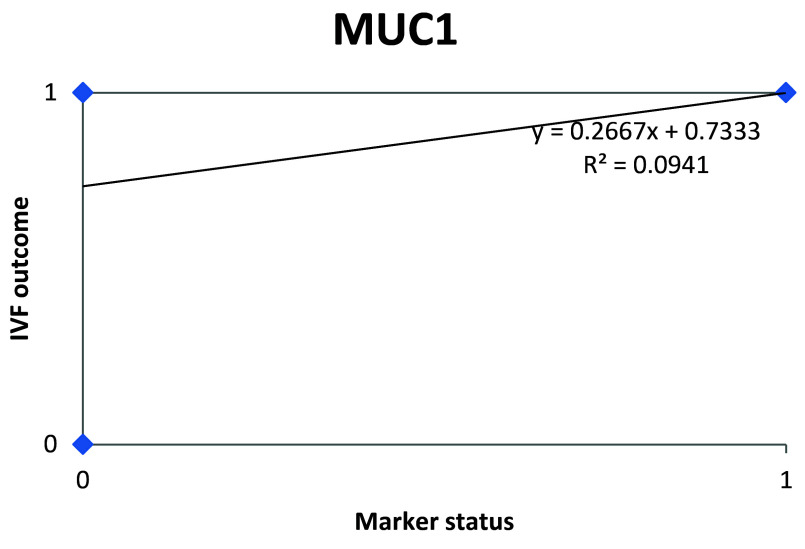
Scatter plot showing correlation between MUC1 status and IVF outcome.

Further calculations concluded that MUC1 status thus had a weak correlation with the IVF outcome, which was statistically insignificant ( Figure 7; Table 4).

**Table 4.** Summary of the relationship between immunohistochemical markers and *in vitro* fertilization outcomes.

Marker	Agreement with IVF outcome	Correlation coefficient	Odds ratio	P-value
MUC1	slight	0.31	5.09	0.23
E-cadherin	fair	0.39	7.31	0.21

**Figure 8.**
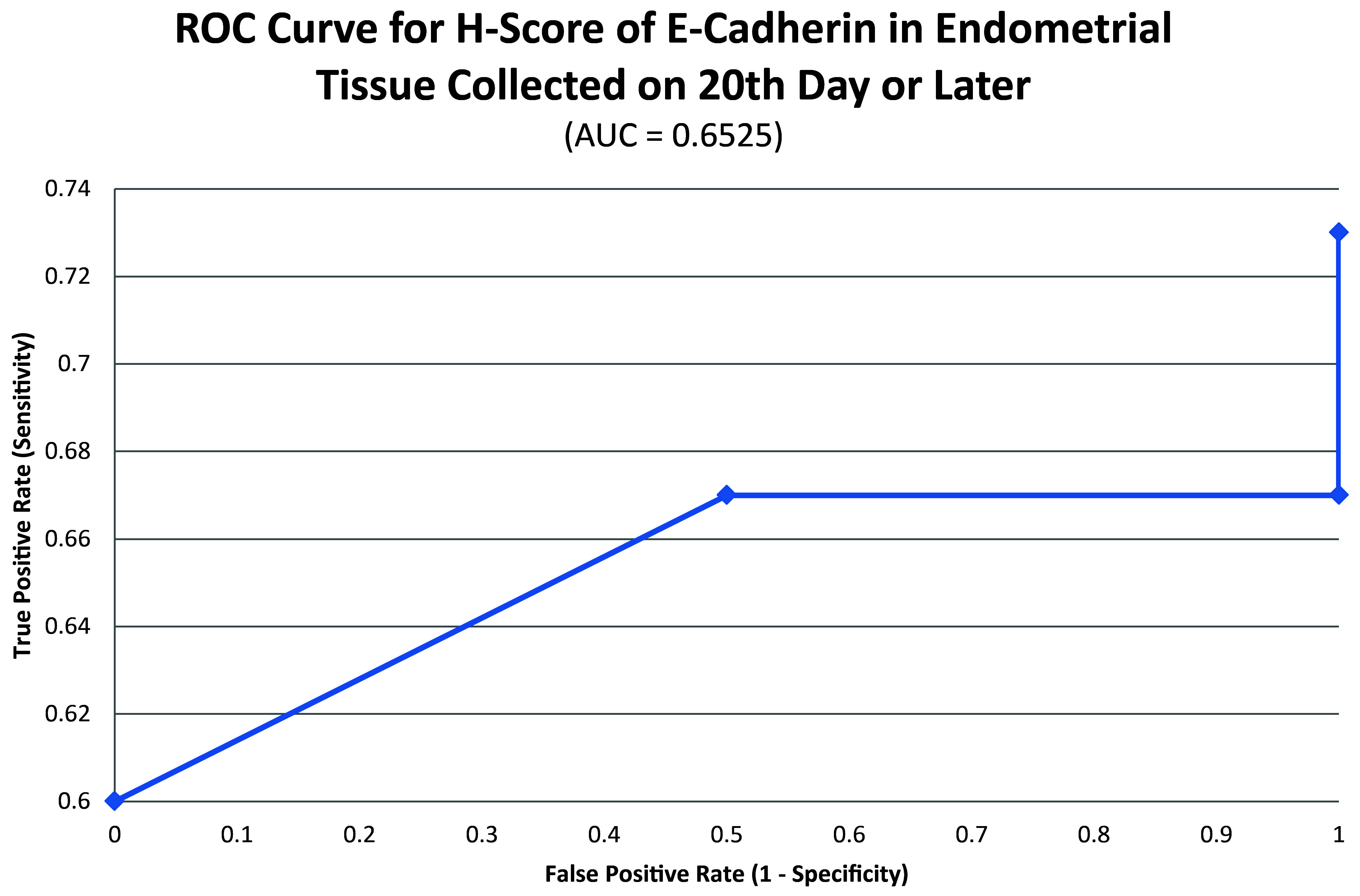
ROC curve for H-score of E-cadherin in endometrial tissue collected on 20 ^th^ chronological day or later.

**Figure 9.**
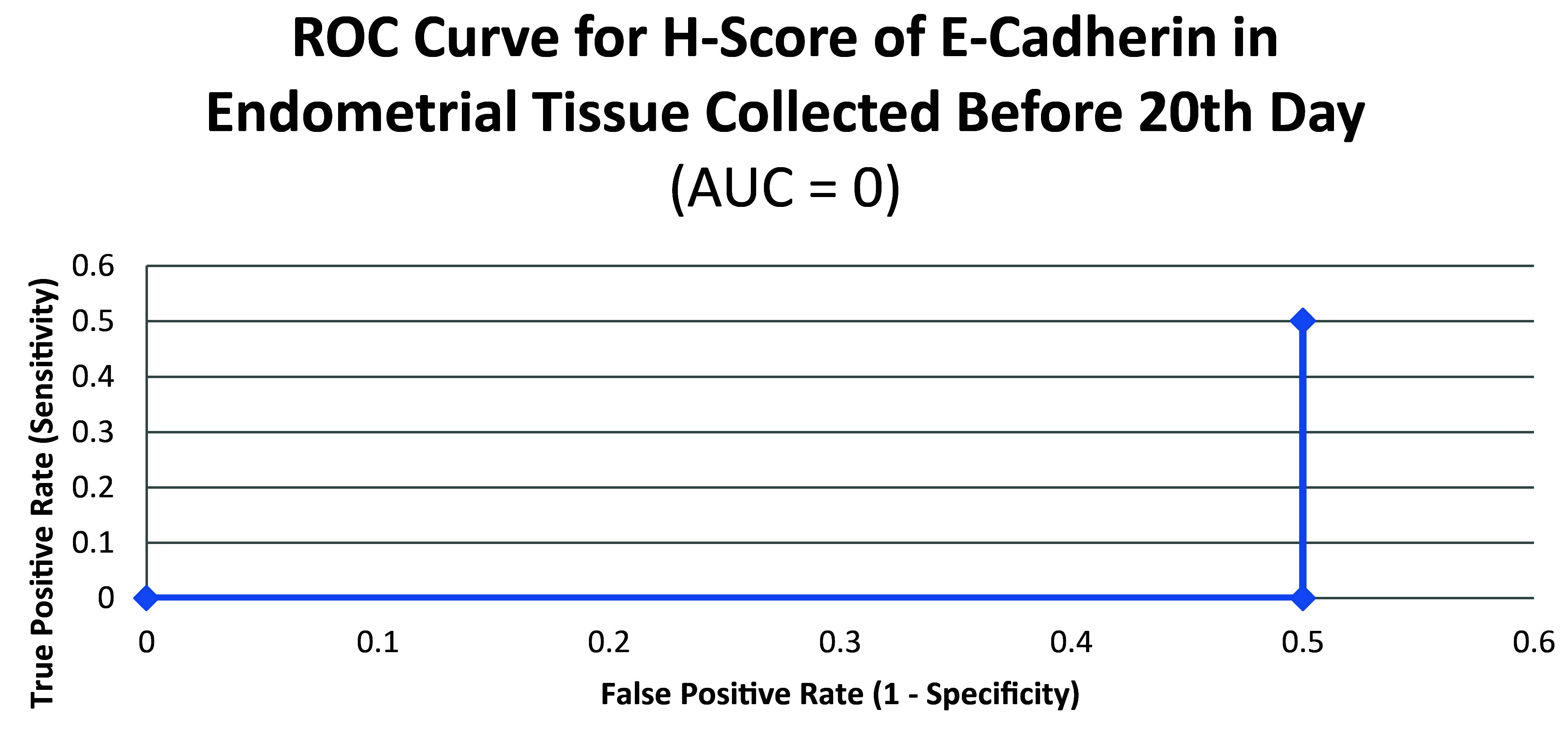
ROC curve for H-score of E-cadherin in endometrial tissue collected before 20 ^th^ chronological day.

**Figure 10.**
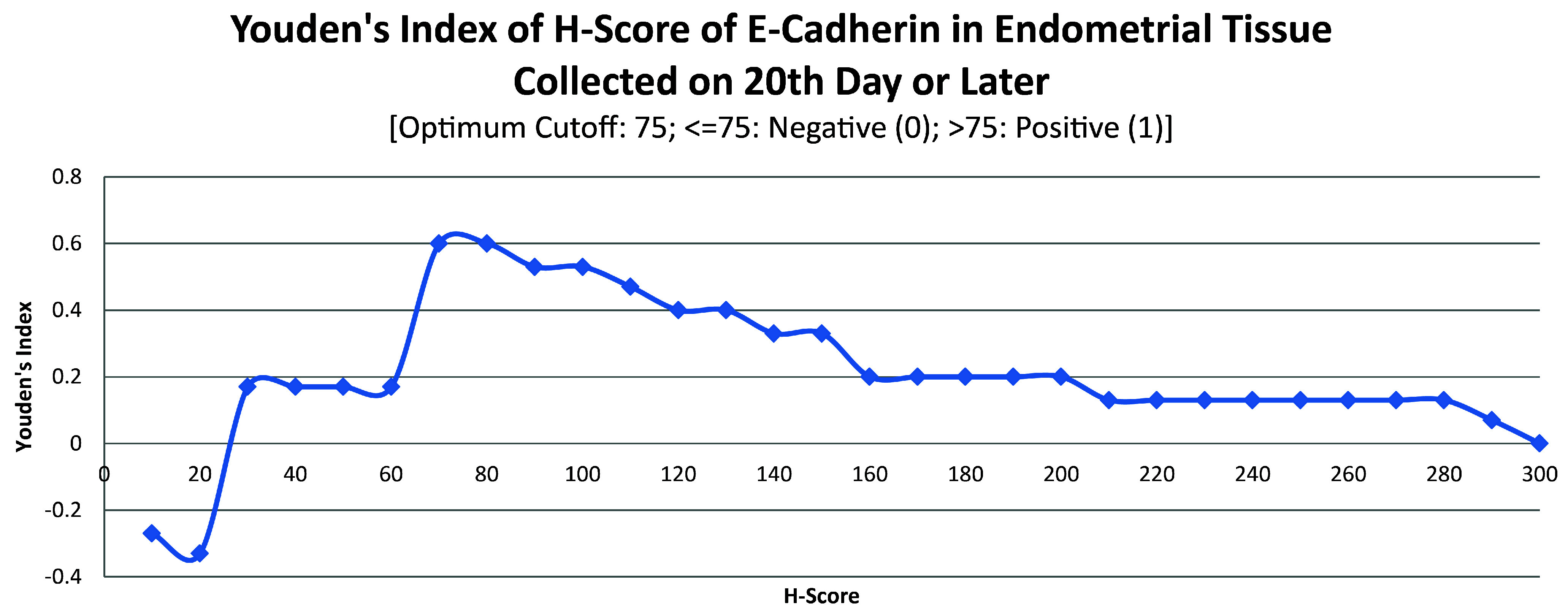
Youden’s index curve for H-score of E-cadherin in endometrial tissue collected on 20 ^th^ chronological day or later.

**Figure 11.**
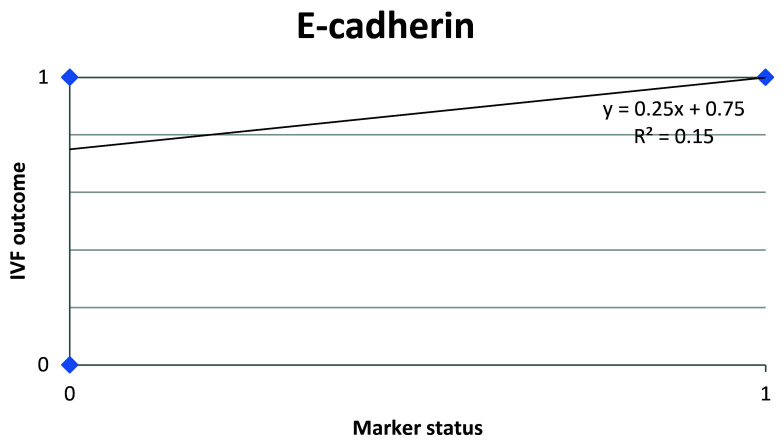
Scatter plot showing correlation between E-cadherin status and IVF outcome.

ROC curve for E-cadherin suggested the existence of optimum cutoff values for samples collected on or after the 20 ^th^ chronological day but not before, since AUC>0 and AUC=0 held for those two instances, respectively ( Figure 8 and Figure 9). The cutoff for samples collected on or after 20 ^th^ day was 75 ( Figure 10). E-Cadherin status is to be considered negative if the H-score is less than or equal to the cutoff; if it is above the cutoff then the status is positive. Subsequent calculations determined that E-cadherin status had a weak correlation with the IVF outcome, which was statistically insignificant ( Figure 11; Table 4).

Various confounding variables were matched between case and control groups in the present study ^24, 25^. Age and BMI are two such anthropometric variables, which were matched in similar studies where comparison was being done between receptive and non-receptive endometrium ^9^. However, ratio of sample size between case and control groups in the present study is about 5:1 which is not shown with most of the studies with similar design where the ratio is almost 1:1. This deviation is in part due to relatively lower IVF success rates that ultimately resulted in fewer number of successes accomplished within the study period. Nevertheless, the imbalance between the two groups in the present study was taken into account statistically by applying adjustments for unequal variance wherever appropriate.

Although endometrial dating by histomorphological features are indeed helpful, it must be noted that the method has its pitfalls. Basal-only sampling, tangential cuts resulting in false crowding of glands, difficulties in detecting spiral arteries and predecidual change, epithelial-stromal discordance, polyps, inflammation etc. often hinder the accuracy of endometrial dating. Also, some of the samples in the present study lack surface epithelium due to the small quantity of the tissue collected, which was somewhat unavoidable (discussed later). All those factors, compounded by subjective nature of the methodology, makes histological dating of endometrium a challenging feat for the pathologist ^26^. Despite the difficulties, best efforts were made to ensure the dating in the present study was as accurate as possible, which included consultation with expert pathologists, checking and double-checking the findings and cross-referencing with multiple authentic sources.

H-score, acronym for ‘histological’ score, was initially developed to quantify immunohistochemical staining of certain tumors but later its use diversified to serve many other arenas of immunohistochemistry including the assessment of endometrial receptivity ^9^. The current study utilized this method to provide a uniform platform for the immunohistochemical markers to be assigned a number that directly reflects its staining characteristics and also makes it more amenable to the robust statistical tools applied subsequently. Due to lack of consensus regarding the roles of immunohistochemical markers and their optimum cutoff values for useful categorization as far as the investigator could search for in English scientific literature, the present study attempted to develop such a scheme tailored for this study from scratch according to the fundamental statistical principles underlying some of the most widely used scoring and categorization systems in diagnostic pathology ^27^.

How should the endometrial expression pattern of MUC1/EMA be in order to facilitate implantation remains an open question since non-congruent results regarding this are frequently found in English scientific literature ^8, 10^. The present study attempted to address the question by incorporating MUC1 into the immunohistochemistry panel but found its correlation with IVF outcome to be weak and statistically insignificant.

Although correlation of E-cadherin H-score with IVF outcome was not statistically significant in the present study, higher H-scores of E-cadherin were found to be more associated with IVF failure which resonates with the findings of other similar studies ^8, 16^. An interesting finding of the present study about E-cadherin was the marked difference of its relative intensity between surface epithelia and glandular epithelia where the surface epithelial staining was almost consistently higher in intensity than glands. A similar finding is noted in a study where such differential staining is appreciated in non-receptive endometrium, except the marker was PGRMC1 and not E-cadherin ^9^. Perhaps further inquiry into such peculiarities would unravel more mysteries in the ever-expanding field of reproductive biology.

Although this study was confined to endometrial samples at a single point of the menstrual cycle which may contribute to non-representative sampling, further research may be designed incorporating endometrial biopsy collection at more than one day of the patient’s menstrual cycle. This might be achieved by iatrogenic thickening of endometrium by progesterone and/or luteinizing hormone administration, preferably six months to one year prior to the IVF embryo transfer cycle to avoid possible complications in implantation process as practiced in case of ERA ^9^. Besides the prediction of IVF outcome, this modified technique may provide necessary information to calculate the WOI for individual patient that might be used to transfer embryo during IVF, thus improving the odds of IVF success. However, hormone-induced changes in immunohistochemical expression of the markers would have to be taken into account if that method is followed ^26^.

Due to ethical and methodological constraints, the amount of endometrial tissue obtained by scratch procedure was generally less than that of routine endometrial curettage. This added to the difficulty in endometrial dating and in appreciating the potential heterogeneous nature of endometrial tissue. One sample had to be kept out of the study pool simply due to sample inadequacy. Moreover, endometrial sample had to be collected just once per patient, thus eliminating the possibility to evaluate samples at more than one point in a menstrual cycle for a patient. Hormonal influence on endometrial tissue may alter the morphological and immunohistochemical findings ^9, 26^. Therefore in the present study, samples were collected prior to commencement of hormone treatment. Since the endometrial ‘scratch’ biopsy procedure was performed on each of the patients of both case and control groups, any possible effect of the procedure itself would have been cancelled out between the two groups. Since this study was partly motivated by an attempt to complement the genomic approach for IVF outcome prediction known as ERA, it would be ideal to be able to cross-validate the findings by ERA of each of the samples. However, financial and time constraints prevented the investigators to pursue this aspect.

It is anticipated that the study will be used as a guide for further research and will provide essential information to design a low-cost alternative diagnostic tool to ERA for predicting IVF failure as well as individualized WOI. Thus, it may help reduce the financial and associated burdens of women undergoing IVF or other infertility treatment.

## Ethical statement

Ethical approval was obtained from the Institutional Review Board of Bangabandhu Sheikh Mujib Medical University (approval no. BSMMU/2017/1576). Written informed consent for publication of the patients’ details was obtained from the patients.

## Data availability

The underlying and extended data is publicly available on Open Science Framework: MUC1 and E-cadherin immunohistochemistry of endometrium cannot predict the outcome of *in vitro* fertilization: A case-control study, https://doi.org/10.17605/OSF.IO/UAMV4 ^28^.

### Underlying data

- Supplementary_material_1: Master datasheet [BMI = Body Mass Index; Menstrual history: 0 = Regular, 1 = Infrequent; IVF = In vitro fertilization; Endometrial phase: 0 = Secretory; 1 = Proliferative; Ep. = Epithelium; St. = Stroma; Pregnancy outcome: 0 = Success/conceived by IVF, 1 = Failure/did not conceive by IVF; Previous IVF outcome: 0 = No H/O IVF, 1 = Previous one failure; FSH = Follicle stimulating hormone; LH = Luteinizing hormone.]
- Raw data for Figure 3– Figure 11
- Digital microscopic slide files are available on request from the corresponding author ( saumitra1880@yahoo.com). As these files are large in size (500MB–1GB each) they are not available on OSF.

### Extended data

- Supplementary_material_2: Proof of internal consistency (Measurement of interobserver variability by Chronbach's Alpha)
- Supplementary_material_3: Data analysis regarding MUC1 to calculate its correlation with IVF outcome and odds ratio along with P value (Cohen's kappa correlation or degree of agreement)
- Supplementary_material_4: Data analysis regarding E-cadherin to calculate its correlation with IVF outcome and odds ratio along with P value (Cohen's kappa correlation or degree of agreement)
- Supplementary_material_5: Patient consent form used in the study in both Bangla and English
- Supplementary_material_6: Routine histology lab protocol for Hematoxylin and Eosin stain used in the study
- Supplementary_material_7: Immunohistochemistry lab protocol for MUC1/EMA and E-cadherin antibody markers used in the study
- Supplementary_material_8: Ethical approval from Institutional Review Board (IRB) of Bangabandhu Sheikh Mujib Medical University (BSMMU) allowing the authors to conduct the study
- Supplementary_material_9: Memorandum of understanding (MOU) between CARe and Department of Pathology, BSMMU

Data are available under the terms of the Creative Commons Zero "No rights reserved" data waiver (CC0 1.0 Public domain dedication).
