# Supplementary material for: MUC1 and E-cadherin immunohistochemistry of endometrium cannot predict the outcome of in vitro fertilization: A case-control study
Source: F1000Res. 2019 Feb 6;8:162. [Version 1] doi: 10.12688/f1000research.17929.1 (PMC13193227; doi:10.12688/f1000research.17929.1)
Supplement: Peer review history

# Reviewer report: Reviewer response for version 1

**DOI:** 10.5256/f1000research.19607.r473870

**Published:** 2026-04-28

**Authors:** Petronela Naghi

**Affiliations:** 1 Calla—Infertility Diagnostic and Treatment Center, Oradea, Romania

The material captures the importance of the endometrium in achieving a pregnancy. The dialogue between the embryo and the endometrium is vital for a successful embryo transfer. The importance of MUC and Ecadherin is emphasized in the text, but the number of samples is small - 17, and the number for the control is 4, without differentiation based on age, and there are few cases to validate the method.

Is the work clearly and accurately presented and does it cite the current literature?

Yes

If applicable, is the statistical analysis and its interpretation appropriate?

I cannot comment. A qualified statistician is required.

Are all the source data underlying the results available to ensure full reproducibility?

Yes

Is the study design appropriate and is the work technically sound?

Yes

Are the conclusions drawn adequately supported by the results?

Yes

Are sufficient details of methods and analysis provided to allow replication by others?

Yes

Reviewer Expertise:

embryology, citology, laboratory medicine

I confirm that I have read this submission and believe that I have an appropriate level of expertise to confirm that it is of an acceptable scientific standard, however I have significant reservations, as outlined above.

# Reviewer report: Reviewer response for version 1

**DOI:** 10.5256/f1000research.19607.r44200

**Published:** 2019-03-15

**Authors:** Indira Hinduja

**Affiliations:** 1 Jaslok Hospital & Research Centre, Mumbai, Maharashtra, India

In the study, the authors evaluated the expression of MUC1 and E-cadherin by immunohistochemistry to determine their role in endometrial receptivity and correlated with the IVF outcome. The study was performed to develop cost effective laboratory investigation as opposed to expensive ERA. However, they did not find statistically significant correlation in case of both the markers. The manuscript is well written in different sections of introduction, methods, results and discussion. However, following are the comments related to the study design and conclusion of the study:

- In the “procedure performed at CARe’’ section, it has been stated that ‘The procedure was performed by a competent physician on the 21 ^st^ day of natural cycle or on a day accordingly adjusted for irregular, longer or hormone assisted cycle immediately before the commencement of IVF cycle’.

Please explain following points:

1. In case of natural cycle, whether the ovulation was detected by follicular monitoring or LH to determine the window of implantation e.g. LH+6/7?
2. In case of women with irregular menstrual cycle, or hormone assisted cycle, how the adjustment was done? which day was considered for endometrial receptivity?
3. In the case group (n=17), how many of the cases were included from natural cycle and how many were adjusted hormonally?
4. Whether the authors have compared these two groups separately?
5. In the study, the patients were selected as cases or controls based on their IVF outcome. Please mention whether all the cases and controls are given similar regimen of hormones or same the hormonal protocol?
6. Whether the patients with PCOS, endometriosis and other hormonal abnormalities were excluded?  
7. In table 1, it has been stated that endometrial biopsy was taken on Day 12 of the menstrual cycle for four patients (which are from the control group). It means that in controls, the endometrial biopsy was taken during proliferative phase. To have comparison of the women with IVF success and women with IVF failure, the endometrial biopsy should be on same day i.e during the window of implantation especially when authors aim to study the endometrial receptivity based on IVF outcome. Please explain this important point in detail.
8. As mentioned in the last paragraph of discussion, please explain how the present study results can be used to predict individualized WOI?
9. Whether the tissue samples stored in trizol or RNA later? Can the markers be correlated with mRNA levels (gene expression levels)?
10. Based on the previous reports, hundreds of genes are involved in the receptivity of endometrium. For instance ERA predicts the day of personalized ET based on m RNA levels of 238 genes. Therefore, it would be difficult to predict the IVF outcome based on only 2 genes. The panel of some of the genes needs to be performed by IHC to achieve the scientific conclusion of designing the low cost alternative diagnostic tool like ERA.  

With respect to the study outcomes are concerned i.e. development of cost-effective immunohistochemistry based test to be a predictor of IVF outcome:

1. The sample size of the study is low
2. The ratio of cases Vs Controls is low (17Vs4)
3. The study should have a third group of healthy fertile women as a reference group which would be the actual control group comparing fertile (natural) Vs infertile (IVF)Vs Fertile (IVF) women

I recommend the manuscript for indexing only after the satisfactory explanation of above queries.

Is the work clearly and accurately presented and does it cite the current literature?

Yes

If applicable, is the statistical analysis and its interpretation appropriate?

I cannot comment. A qualified statistician is required.

Are all the source data underlying the results available to ensure full reproducibility?

Yes

Is the study design appropriate and is the work technically sound?

Partly

Are the conclusions drawn adequately supported by the results?

Partly

Are sufficient details of methods and analysis provided to allow replication by others?

Yes

Reviewer Expertise:

IVF, reproductive biology, endometrial receptivity

I confirm that I have read this submission and believe that I have an appropriate level of expertise to confirm that it is of an acceptable scientific standard, however I have significant reservations, as outlined above.

# Reviewer report: Reviewer response for version 1

**DOI:** 10.5256/f1000research.19607.r44393

**Published:** 2019-02-27

**Authors:** Antonio Simone Laganà

**Affiliations:** 1 Department of Obstetrics and Gynecology, Filippo Del Ponte Hospital, University of Insubria, Varese, Italy

I read with great interest the Manuscript titled “MUC1 and E-cadherin immunohistochemistry of endometrium cannot predict the outcome of in vitro fertilization: A case-control study” (F1R-VER19607-R), which falls within the aim of F1000Research.

 In my honest opinion, the topic is interesting enough to attract the readers’ attention. Methodology is accurate and conclusions are supported by the data analysis. Nevertheless, authors should clarify some points and improve the discussion citing relevant and novel key articles about the topic.

 Authors should consider the following recommendations:

- Manuscript should be further revised by a native English speaker.
- Was this study registered? I could not find any information about this point.
- Embryo transfer is a key stage in IVF, in which the quality of performance determines the outcome. According to recent evidence, transvaginal ultrasound guidance of the transfer significantly increases the percentage of pregnancies per transfer, both in the general population and in the reference population, compared with transfers performed under transabdominal ultrasound guidance. Authors should add few lines about the topic, referring to: Cozzolino et al.(2018) ^1^; Larue et al.(2017) ^2^.
- Recent and robust data suggested that endometrial scratch injury performed once, may improve clinical pregnancy and ongoing pregnancy rates in IUI/IVF cycles. I invite authors to discuss these data (refer to: Vitagliano et al. (2018) ^3^; Vitagliano et al.(2018) ^4^; Vitagliano et al. (2019) ^5^), in the light of available evidence.

Is the work clearly and accurately presented and does it cite the current literature?

Yes

If applicable, is the statistical analysis and its interpretation appropriate?

Yes

Are all the source data underlying the results available to ensure full reproducibility?

Yes

Is the study design appropriate and is the work technically sound?

Yes

Are the conclusions drawn adequately supported by the results?

Yes

Are sufficient details of methods and analysis provided to allow replication by others?

Yes

Reviewer Expertise:

Reproductive medicine

I confirm that I have read this submission and believe that I have an appropriate level of expertise to confirm that it is of an acceptable scientific standard.

## References

[1] : Ultrasound-guided embryo transfer: summary of the evidence and new perspectives. A systematic review and meta-analysis. *Reprod Biomed Online* .2018;36(5) : 10.1016/j.rbmo.2018.01.015 524-542 10.1016/j.rbmo.2018.01.015 29576332

[2] : Transvaginal ultrasound-guided embryo transfer in IVF. *J Gynecol Obstet Hum Reprod* .2017;46(5) : 10.1016/j.jogoh.2017.02.015 411-416 10.1016/j.jogoh.2017.02.015 28428123

[3] : Endometrial scratch injury before intrauterine insemination: is it time to re-evaluate its value? Evidence from a systematic review and meta-analysis of randomized controlled trials. *Fertil Steril* .109(1) : 10.1016/j.fertnstert.2017.09.021 84-96.e4 10.1016/j.fertnstert.2017.09.021 29175068

[4] : Does endometrial scratching really improve intrauterine insemination outcome? Injury timing can make a huge difference. *J Gynecol Obstet Hum Reprod* .2018;47(1) : 10.1016/j.jogoh.2017.11.001 33-34 10.1016/j.jogoh.2017.11.001 29127042

[5] : Endometrial scratching for infertile women undergoing a first embryo transfer: a systematic review and meta-analysis of published and unpublished data from randomized controlled trials. *Fertil Steril* .2019; 10.1016/j.fertnstert.2018.12.008 10.1016/j.fertnstert.2018.12.008 30683590
